# The Effect of Introducing a Smaller and Lighter Basketball on Female Basketball Players’ Shot Accuracy

**DOI:** 10.2478/v10078-012-0014-8

**Published:** 2012-04-03

**Authors:** Nadja Podmenik, Bojan Leskošek, Frane Erčulj

**Affiliations:** 1 University of Ljubljana, Faculty of Sport, Ljubljana, Slovenia.

**Keywords:** basketball, women, size 6 basketball, playing efficiency

## Abstract

Our study examined whether the introduction of a smaller and lighter basketball (no. 6) affected the accuracy of female basketball players’ shots at the basket. The International Basketball Federation (FIBA) introduced a size 6 ball in the 2004/2005 season to improve the efficiency and accuracy of technical elements, primarily shots at the basket. The sample for this study included 573 European female basketball players who were members of national teams that had qualified for the senior women’s European championships in 2001, 2003, 2005 and 2007. A size 7 (larger and heavier) basketball was used by 286 players in 1,870 matches, and a size 6 basketball was used by 287 players in 1,966 matches. The players were categorised into three playing positions: guards, forwards and centres. The results revealed that statistically significant changes by year occurred only in terms of the percentage of successful free throws. With the size 6 basketball, this percentage decreased. Statistically significant differences between the playing positions were observed in terms of the percentage of field goals worth three points (between guards and forwards) and two points (between guards and centres). The results show that the introduction of the size 6 basketball did not lead to improvement in shooting accuracy (the opposite was found for free throws), although the number of three-point shots increased.

## Introduction

Contemporary elite basketball requires players to have high levels of accuracy in varying conditions during the game (Jovanović-Golubović and Jovanović, 2003). Shooting accuracy is of great importance. The shooting technique differs according to the distance from the basket and depends, to some extent, on the player’s body height and playing position. When shooting at the basket from a distance, a jump shot with a two-leg take off is usually used, generating about 41% of all points in a match (Baloncesto, 1997, in Tang and Shung, 2005). Shots under the basket include different shots with a one-leg take-off. The release angle for shots from a short distance is usually 52– 55°, whereas it is smaller for shots from a longer distance, usually 48–50° (Miller and Bartlett, 1993; 1996; Rojas et al., 2000). The possibility of a deviation from the optimal values is greater if the basketball is in the air for a longer period of time (Karalejić and Jakovljević, 2008). In all playing positions, the longer the distance, the faster the release of the ball (Miller and Bartlett, 1996) and, consequently, the more accurate the shot must be (Jovanović-Golubović and Jovanović, 2003). It should also be noted that basketball is a situational game; the players shoot toward the basket from different positions and in different situations, making either close-range or long-range shots and with the basketball either bouncing off the backboard or not touching it. These shots are more or less hindered by the defensive players. The accuracy of shots at the basket is therefore a complex issue that is affected by many factors. The only exception to this complexity is a free throw, which is executed under much more controlled and stable conditions. The accuracy of a free throw is affected by fewer factors.

The shot at the basket is one of the elements that significantly influences performance in basketball. To execute a shot properly, a player requires good motor abilities, which differ between men and women. One of the most apparent and important gender-related differences in performance in many sports is the ratio between strength and body mass, which skews in favour of men during puberty (DeVries, 1986). A similar issue applies to basketball and, especially, to shots at the basket. The positive effect of strength on shooting accuracy has been corroborated by many authors (Sklerynk and Bedingfield, 1985; Sherwood et al., 1988; Tang and Shung, 2005; Justin et al., 2006).

In 1935, Dittebrant suggested the introduction of a smaller and lighter basketball for female players (Dittebrant, 1935, in Pitts and Semenick, 1988). The author stated that the standard basketball was too heavy and too large for women, and that women’s palms were too small to efficiently control the ball. A smaller and lighter basketball (size 6) for women was introduced in the United States’ Women’s Professional Basketball League (WBL) in 1978 (Nissen, 1997). The players’ response to the smaller ball was extremely positive, and it was suggested that this basketball should be used in other competitions in the US. However, Europe deferred a similar decision until the 12^th^ of June 2004, when the Central Board of FIBA amended the Official Basketball Rules. In the 2004/2005 season, FIBA finally introduced a smaller and lighter basketball for female players to be used in all competitions under FIBA’s auspices (Official Basketball Rules, 2004).

The FIBA Official Basketball Rules do not specify the size or mass of the ball; they only specify the range of measurements. The difference between sizes 6 and 7 is 34 mm of circumference, 10.8 mm of diameter and 70 g of mass, all in the middle of the range interval. Consequently, with the introduction of the size 6 basketball, the ratio between the diameter of the ball and the basket (hoop) changed, as did the ratio between the clear area of the basket (ring), the area of the ball (projection to the plane) and the minimum entry angle (angle of incidence) at which the ball passes through the basket. Therefore, it can be concluded, at least theoretically, that it is easier to score with a size 6 basketball (a smaller ball) and that, consequently, the share of field goals would be higher.

The smaller and lighter basketball was introduced to make women’s basketball more interesting to spectators because female players tend to shoot from longer distances more often than men do, and they generally have more accurate control of the ball when shooting (Logan, 1978). The aim of our study was to verify whether the introduction of a smaller and lighter ball affected female players’ shooting accuracy at the basket, especially for long range shots (three-point shots) and free throws. Another area of interest was whether the number of shots from a long distance (i.e. three-point shots) increased.

Although female basketball players in Europe have used the size 6 basketball for six years (and it has been used for much longer in the United States), this study is the first to examine this subject. We have found no previous work in Europe or elsewhere that has used playing statistics to determine whether the introduction of a smaller and lighter ball is justified and reasonable for female basketball players in terms of greater playing efficiency.

## Material & Methods

The sample included all female basketball players who were members of national teams that had qualified for the senior women’s European championships in 2001 (France), 2003 (Greece), 2005 (Turkey) and 2007 (Italy). In these championships, 286 players used a size 7 basketball in 1,870 matches and 287 players used a size 6 basketball in 1,966 matches. The total playing time with size 7 and size 6 basketballs was 36,873 and 37,698 min, respectively.

The players were divided into three groups according to their playing positions: guards, forwards and centres. The analysis of the percentage of field goals included only those players who took a one-, two- or three-point shot at the basket at least five times. This criterion was applied to avoid extreme cases that could affect the results. Thus, 96.8% of all players were included for the two-point shot, 61.9% for the three-point shot and 77.7% for free throws. This criterion was not applied for binary logistic regression.

Our study included players of 12 teams from each European Championship. Accordingly, all the teams from the 2001, 2003 and 2005 European Championships were included, whereas in 2007, the best 12 teams were included.

*The analysis of the statistical data by* playing position included the following variables: percentage of field goals worth two points, percentage of field goals worth three points, percentage of free throws scored, number of two-point shots, number of three-point shots and number of free throws.

The data were acquired from FIBA’s official website (www.fiba.com), where the official basketball statistics are published for all of the above mentioned European championships. These data were analysed using the PASW 18.0.3 software and Microsoft Excel. The results were presented using descriptive statistics and diagrams. For each dependent variable, a two-way analysis of variance (year, playing position) was used. If significant effects were found, post hoc testing was performed, applying Tukey’s HSD for multiple comparisons. The statistical significance of all tests was set at p < 0.05. A binary logistic regression revealing shots at the basket from different positions – where the year of the EC and the playing position were independent factors, and players were a random factor – was performed in R 2.12.0. The glmmPQL function was used. The reference categories of factors were the year 2001 and the playing position of a guard.

## Results

The percentage of all two and three point field goals did not change substantially by championship. In terms of free throws, the highest percentage occurred at the 2001 EC and declined gradually over the following ECs. Likewise, there were no changes in the number of two point shots. The number of three point shots was higher at the 2005 and 2007 ECs, where a smaller and lighter basketball was used. Regarding free throws, the number of shots and field goals decreased at the 2005 and 2007 ECs.

**Figure 1:**
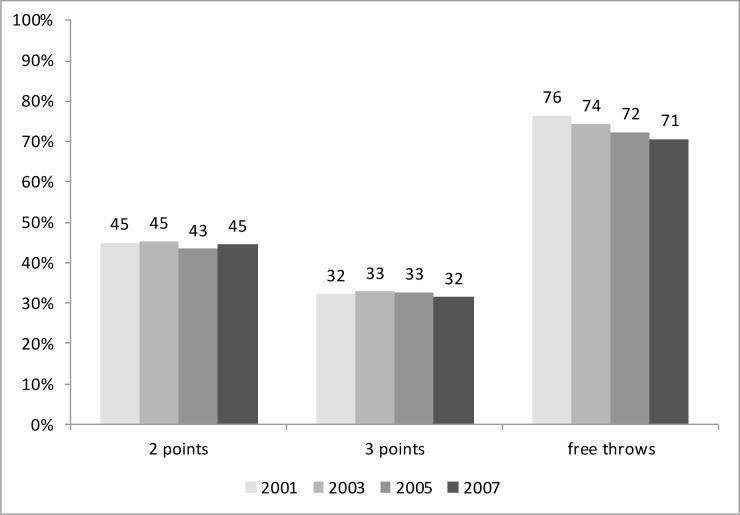
Percentage of field goals by EC

Similar ratios were observed in the percentage of all field goals (Figure 1). The two and three point shots had similar percentages, whereas the value of free throws decreased over time.

The number of three point shots (Table 2) and free throws at the European Championship differed in an inverse proportion. As a rule, the number of three point shots increased over time, whereas the number of successful free throws decreased. This change is characteristic for both guards and forwards, whereas it occurred only in case of free throws for the centres.

**Figure 2:**
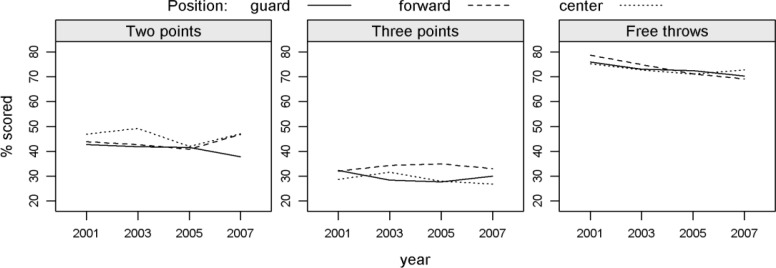
Average percentage of scored one-, two- and three-point shots by playing position

In all ECs, the largest share of scored two point shots was recorded by centres (Figure 2). This percentage was only slightly higher than the percentage for forwards in the years when the size 6 basketball was used. The guards had a similar percentage as the forwards, but this percentage decreased in the 2007 EC. In terms of three point shots, in the two-way (year, playing position) ANOVA, forwards recorded a statistically significantly higher percentage of field goals than guards (Tukey’s HSD post-hoc test, p=0.03). In all ECs, forwards had the highest percentage of three-point shots, whereas in the case of guards and centres, this percentage varied by EC. Statistically significant (p=0.03) differences between years occurred only in the percentage of free throws scored, although this percentage decreased in the 2005 and 2007 ECs when the size 6 basketball was used.

Similar results were obtained with a binary logistic model with the tournament year and player position as fixed factors and the player as a random factor (Table 3). In this analysis, all players and all shots were included (in contrast with the ANOVA, in which players with fewer than five shots were excluded from the analysis). Compared with the reference player position (guard), centres were found to have higher odds (AOR=1.24) of scoring two point shots. In three-point shots, the odds for forwards were significantly higher (AOR=1.18) than the odds for guards. Compared to EC 2001, the accuracy of free throws was significantly lower in 2005 (AOR=0.81) and, especially, in 2007 (AOR=0.72).

## Discussion

The results of the study show no substantial differences in terms of the percentage of two-point field goals when using the old and new basketballs. The centres had the highest successful shooting percentages, and they were statistically significantly higher than those of the guards. This finding is expected because the centres, on offence, most often decide to take close-range shots (from under the basket). This shot is generally easier to score than a shot from a greater distance. It is interesting that in 2005 (the year the size 6 basketball was used for the first time), the percentage of the centres’ field goals dropped considerably, to only 42.1%. This decrease could be a consequence of the centres’ inability to rapidly adjust to the smaller and lighter ball compared to the other playing positions.

When analysing the three point shot, the figures pertaining to the centres must be explained cautiously because centres very rarely decide to take a shot from such a distance. An increase was observed in the number of such shots made by guards and forwards. This is the only change that was expected with the introduction of the smaller and lighter ball. In her doctoral thesis, Pitts (1985) stated that the use of the smaller and lighter ball boosts female players’ self-confidence, which is why players decide more often to take a three point shot. This finding was also confirmed in our research, although it would be expected that self-confidence would be primarily enhanced by successful three point shots. In our case, the percentage of field goals worth three points did not increase with the size 6 basketball.

In basketball, a free throw is executed under relatively controlled and stable conditions. The player takes unhindered shots, always from the same distance. The accuracy of the shot is not influenced by as many factors as field shots. We believe that this is why this type of shot demonstrates the most “pure” effect of introducing the size 6 basketball on the accuracy of shooting at the basket. The results show that in all playing positions (guards, forwards and centres), the introduction of the size 6 basketball resulted in a statistically significant decrease in the percentage of free throws scored, rather than an increase, as expected. It seems that the distance from which the female basketball players made free throw shots was not large enough that the slightly heavier size 7 basketball would become too heavy and impair shooting precision. These results are probably not due to the effect of the change in basketball size on shooting technique. Okazaki and Rodacki (2005) found that the size of the basketball does not affect children’s coordination or shooting technique. Therefore, it can be concluded that no changes occurred in the shooting technique of our sample of female basketball players. Nevertheless, the result indicating the diminished accuracy of the execution of free throws with the smaller and lighter ball is quite surprising.

Given the characteristics of the male and female body – primarily the gender-related difference in strength – the introduction of a smaller and lighter ball is an understandable and expected change. Despite all reasons in favour of introducing the size 6 basketball, the use of the smaller and lighter ball clearly does not improve shooting accuracy. It may be concluded that shooting accuracy is a complex issue that depends on a large number of factors, only some of which have been addressed in our study. This is particularly true for cases in which shooting accuracy is established for field goals that are executed from different positions and in different situations. The use of the smaller and lighter basketball in women’s basketball affects not only shooting accuracy but also other elements, such as dribbling, passing, and better handling of the ball, which were not tested in our study. Therefore, the results of this study cannot be generalised, and the positive effect of the introduction of the size 6 basketball on female basketball players cannot be absolutely negated. However, the findings of this study reveal that the introduction of the new ball for female basketball players may not have been fully justified and reasonable. In the future, the competent bodies that make such important amendments to the rules should base their decisions on research findings and verify these decisions in practice. This method could help to prevent criticism that these decision-making bodies are conceding to pressure from sports equipment manufacturers and their market interests.

## Figures and Tables

**Table 1:** Basic characteristics of the sample – index (2001=100)

Year	**2001**	**2003**	**2005**	**2007**
No. of female basketball players	100	101	101	101
No. of matches	100	99	103	107
Average (min)	100	98	100	103
Total (min)	100	100	100	104

**Table 2:** Average values of shots and goals by playing position

	
		2 points		3 points		free throws	

Position	year	shots	goals	shots	goals	shots	goals
guard	2001	22.9	9.7	11.1	3.6	14.1	10.7
	2003	20.8	8.8	13.8	4.4	11.9	8.8
	2005	22.0	9.3	13.5	4.0	9.2	6.6
	2007	21.3	8.7	14.3	4.3	9.8	6.6

forward	2001	29.8	12.8	8.2	2.8	13.2	10.3
	2003	30.3	13.3	11.3	3.8	13.5	10.3
	2005	32.8	14.3	14.1	5.2	10.3	7.3
	2007	30.8	13.7	14.0	4.7	9.1	6.5

centre	2001	37.3	18.2	3.3	.8	16.0	12.1
	2003	35.7	17.4	3.9	1.3	16.9	12.3
	2005	36.8	16.2	3.7	1.0	10.4	7.6
	2007	35.5	16.9	3.6	1.0	13.3	9.7

Total	2001	29.6	13.3	7.7	2.5	14.4	11.0
	2003	28.4	12.9	10.0	3.3	13.9	10.3
	2005	30.1	13.0	11.0	3.6	9.9	7.2
	2007	28.8	12.8	11.1	3.5	10.6	7.5

**Table 3:** Results of binary logistic regression with year of tournament and position as fixed factors, players as a random factor and success of shots as a response

	
	Adjusted odds ratio (95% confidence interval)		

	2 points	3 points	free throws
Year: 2003	0.99 (0.90–1.08)	1.03 (0.87–1.22)	0.89 (0.76–1.04)
Year: 2005	0.94 (0.85–1.03)	1.01 (0.85–1.20)	**0.81 (0.68–0.97)**
Year: 2007	0.96 (0.87–1.06)	0.97 (0.81–1.15)	**0.72 (0.61–0.85)**
Position: forward	1.08 (0.98–1.20)	**1.18 (1.04–1.35)**	1.02 (0.86–1.22)
Position: centre	**1.24 (1.12–1.37)**	0.87 (0.70–1.07)	1.03 (0.86–1.22)
